# AITRL, an evolutionarily conserved plant specific transcription repressor regulates ABA response in Arabidopsis

**DOI:** 10.1038/s41598-020-80695-2

**Published:** 2021-01-12

**Authors:** Yanxing Ma, Hainan Tian, Rao Lin, Wei Wang, Na Zhang, Saddam Hussain, Wenting Yang, Chen Zhang, Ganghua Zhou, Tianya Wang, Shucai Wang

**Affiliations:** 1 Laboratory of Plant Molecular Genetics and Crop Gene Editing, School of Life Sciences, Linyi University, Linyi, China; 2 Key Laboratory of Molecular Epigenetics of MOE, Northeast Normal University, Changchun, China

**Keywords:** Molecular biology, Physiology, Plant sciences

## Abstract

Expression of stress response genes can be regulated by abscisic acid (ABA) dependent and ABA independent pathways. Osmotic stresses promote ABA accumulation, therefore inducing the expression of stress response genes via ABA signaling. Whereas cold and heat stresses induce the expression of stress response genes via ABA independent pathway. ABA induced transcription repressors (AITRs) are a family of novel transcription factors that play a role in ABA signaling, and *Drought response gene* (*DRG*) has previously been shown to play a role in regulating plant response to drought and freezing stresses. We report here the identification of DRG as a novel transcription factor and a regulator of ABA response in Arabidopsis. We found that the expression of *DRG* was induced by ABA treatment. Homologs searching identified AITR5 as the most closely related Arabidopsis protein to DRG, and homologs of DRG, including the AITR-like (AITRL) proteins in bryophytes and gymnosperms, are specifically presented in embryophytes. Therefore we renamed DRG as AITRL. Protoplast transfection assays show that AITRL functioned as a transcription repressor. In seed germination and seedling greening assays, the *aitrl* mutants showed an increased sensitivity to ABA. By using qRT-PCR, we show that ABA responses of some ABA signaling component genes including some *PYR1-likes* (*PYLs*), *PROTEIN PHOSPHATASE 2Cs* (*PP2Cs*) and *SUCROSE NONFERMENTING 1 (SNF1)-RELATED PROTEIN KINASES 2s* (*SnRK2s*) were reduced in the *aitrl* mutants. Taken together, our results suggest that AITRLs are a family of novel transcription repressors evolutionally conserved in embryophytes, and AITRL regulates ABA response in Arabidopsis by affecting ABA response of some ABA signaling component genes.

## Introduction

As one of the five classic plant hormones, abscisic acid (ABA) regulates multiple aspects of plant growth and development, such as seed development, seed germination, bud dormancy and stomatal movement^1–5^. Most importantly, ABA is a key hormone in regulating plant responses to abiotic environmental stresses including drought, salinity, cold and heat^3–8^. Regulation of plant abiotic stress responses by ABA is usually related to osmotic stresses caused by drought and other water limiting conditions, which can promote ABA accumulation^1,9–11^, whereas ABA signal transduction lead to the activation/repression of stress related genes, thereby affecting plants response to abitotic stresses^3,6–8,12–14^.

ABA signaling is mediated by a few key regulators including the Pyrabactin resistance 1/PYR1-likes/Regulatory component of ABA receptors (PYR1/PYLs/RCARs) receptors^15–17^, the A-group PROTEIN PHOSPHATASE 2Cs (PP2Cs) phosphatases^18,19^, the SUCROSE NONFERMENTING 1 (SNF1)-RELATED PROTEIN KINASES 2s (SnRK2s) protein kinases^20^, and the ABA-responsive element binding protein/ABRE-binding factor/ABA INSENSITIVE 5 (ABF/AREB/ABI5)-type basic region leucine zipper (bZIP) transcription factors^21,22^.

At the absence of ABA, PP2Cs phosphatases interact with, and inhibit the function of SnRK2s kinases. Perception of ABA molecules by PYR1/PYLs/RCARs receptors allows them to interact with PP2Cs, therefore lead to the release and self-activation of SnRK2s. Activated SnRK2s are able to phosphorylate and activate ABF/AREB/ABI5-type bZIP transcription factors, resulting in the activation/repression of ABA response genes, and plant responses to abiotic stresses^4,5,8,13,14,18–20,23^.

The expression of hundreds and thousands of stress related genes is regulated by ABA ^14,23,24^. However, functions of many of them remained largely unknown. In an attempt to identify novel players in ABA signaling transduction, we previously identified ABA induced transcription repressors (AITRs) as a novel family of transcription factors^8^. We found that the expression of *AITRs* is up-regulated by ABA, and AITRs play a role in regulating ABA signaling transduction^8^. Most importantly, we found that AITRs are conserved in angiosperms, and may have been evolved from AITR-like proteins in bryophytes and gymnosperms^8^.

Regulation of stress related genes by ABA affects plant responses to abiotic stresses^1,4,5,9–11^. It has been shown that the conserved PyACGTGG/TC ABRE in the promoter regions of ABA regulated stress related genes is the major *cis*-element for binding of ABF/AREB/ABI5-type bZIP transcription factors^11,25^. However, expression of some stress related genes can be regulated in an ABA indendent payway. Analysis of the promoter regions of these genes has identified the conserved A/GCCGAC dehydration-responsive element (DRE) *cis*-element, as a binding site for APETALA 2/ETHYLENE RESPONSE FACTORS (AP2/ERF) transcription factor DREB1 and DREB2, whose expression was induced by cold and osmotic stress, respectively^26^. In addition, Some NAM, ATAF, and CUC (NAC) transcription factors can also regulate stress related gene expression in an ABA independent pathway via binding directly to the CACG NAC recognition sequence (NACR) *cis*-element in the promoter regions of some stress related genes^27^.

It has been previously reported that the expression of *Drought response gene* (*DRG*) was induced by drought treatment, and DRG is involved in the regulation of plant responses to drought and freezing stresses^28^. We found in this report that the expression of *DRG* was induced by ABA, and the most closely related protein to DRG in Arabidopsis is AITR5. However, DRG is more closely related to the AITR-like (AITRL) proteins in bryophytes and gymnosperms, we therefore renamed it as AITRL. We found that AITRL is involved in the regulation of plant response to ABA, but have opposite functions with AITRs. We also found that AITRLs may represent a family of novel transcription repressors, and are evolutionally conserved in embryophytes.

## Materials and methods

### Plant materials and growth conditions

The Columbia-0 (Col-0) wild type Arabidopsis was used for protoplast isolation and plant transformation. The transgenic plants overexpressing *AITRL* were generated by transform the Col wild type plants with the *35S:AITRL* construct. The T-DNA insertion lines of SALK_022729^28^ and SALK_203161were obtained from the Arabidopsis Biological Resource Center (ABRC, Ohio State University, Columbus, OH, USA), and used to identified homozygous *aitrl-1/drg*^28^ and *aitrl-2* mutants, respectively. The ABA-deficient mutant *aba1-5* was obtained from ABRC and used to examine the expression of *AITRL*.

For RNA isolation, seed germination and seedling greening assays, seeds of the Col wild type, the *35S:AITRL* transgenic plants, and the *aitrl* mutants were surface sterilized and sown on plates with solidified 0.5 × MS salts containing 1% sucrose. The plates were kept at 4 °C and in darkness for 2 days, and then transferred into a plant growth room.

For protoplast isolation and plant transformation, seeds of the Col wild type were germinated directly in soil pots and grew in a growth room. The temperature at the growth room was set at 22 °C, and the light/dark cycle at 16 h/8 h with light density at ~ 120 μmol/m^2^/s.

### Bioinformatics analysis of AITRLs

Homologs of AITRL in other plant species were identified by using “Protein Homologs” on Phytozome (https://phytozome.jgi.doe.gov/pz/portal.html#). Full-length amino acid sequences of the AITRs and AITRLs in selected plant species were obtained on phytozome, and used for amino acid sequence alignment by using BioEdit, and for phylogenetic analysis by using “One Click” mode on phylogeny (http://www.phylogeny.fr/simple_phylogeny.cgi) with default settings.

Gene numbers of the *AITRs* and *AITRLs* were obtained by using *AITR5* and *AITRL*, respectively for “Gene Ancestry” assays on phytozome. Average gene number per plant specie was calculated for different catalogs of the angiosperms.

### ABA reatment, RNA isolation and quantitative RT-PCR (qRT-PCR)

To examine the expression of *AITRL* in response to ABA in the Col wild type seedlings, and the expression of ABA signaling component genes in the Col wild type seedlings, the *35S:AITRL* transgenic plants and the *aitrl* mutants, 12-day-old seedlings were treated with 50 μM ABA in darkness for 4 h, frozen in liquid N_2_ and used for RNA isolation. Seedlings treated with methanol were used as a control. To examine the expression of *AITRL* in *aba1-5* mutant, 12-day-old seeding were frozen in liquid N_2_ and used for RNA isolation.

Total RNA was isolated by using an EasyPure plant RNA kit (Transgen Biotech), and 2 μg of the total RNA isolated was subjected to first-strand cDNA synthesis by using an EasyScript First-strand DNA Synthesis Super Mix (TransGen Biotech). Synthesized cDNA was used as templates for RT-PCR or qRT-PCR analysis. The expression of *ACT2* was used as an inner control. The primers used for RT-PCR analysis of *AITRL* are 5′-CAACATATGATAAAGATACTCAACCCCCA-3′ and 5′-CAAGAGCTCCTATCTGCGGTCAGTGGTTG-3′. The primers used for qRT-PCR analysis of ABA signaling component genes were as described previously^8,29,30^.

### Constructs

The LD-VP activator, the Gal4 DNA binding domain (GD) control effector, the NLS-RFP nuclear indicator, and the *LexA-Gal4:GUS* reporter constructs for protoplast transfection were described previously^31–33^. The *35S:GFP* construct was provided by Prof. Zheng-Yi Xu (Northeast Normal University).

To generate the *GD-AITRL* construct for protoplast transfection assays, the full length open reading frame (ORF) sequence of *AITRL* was amplified by RT-PCR as described above for the examination of the ABA response of *AITRL*, and cloned into the *pUC19* vector with a N-terminal GD tag under the control of the *35S* promoter^32,33^. The *GFP-AITRL* construct was generated by replacing the GD tag in the *GD-AITRL* construct with a GFP tag.

To generate the *35S:AITRL* construct for plant transformation, the GD tag in the *GD-AITRL* construct was replaced with a HA tag, and then digested and subcloned into the binary vector *pPZP211*^34^.

### Plant transformation and transgenic plant selection

The Col wild type plants ~ 5-week-old and with several mature flowers were used for plant transformation, and the plants were transformed via *Agrobacterium tumefaciens* GV3101 mediated tansformation by using floral dip method^35^.

To select transgenic plants, T1 seeds were collected and germinated on plates with solidified 50 μg/ml Kanamycin and 100 μg/ml Carbenicillin containing 0.5 × MS salts. To select transgenic plants with a single T-DNA insertion, T2 seeds collected from transgenic T1 plants were germinated on plates with solidified 25 μg/ml Kanamycin containing 0.5 × MS salts. To identify T3 homozygous overexpression plants, T3 seeds collected from T2 plants were selected on plates with solidified 25 μg/ml Kanamycin containing 0.5 × MS salts, and the expression levels of *AITRL* in the transgenic plants were examined by RT-PCR. Two confirmed homozygous overexpression lines were used for the experiments.

### Plasmid DNA isolation, protoplast isolation and transfection

Plasmid DNA of the reporter and the effectors was isolated from transformed *E. coil* cells by using a GoldHi EndoFree Plasmid Maxi Kit (OMEGA BIO-TEK), and the concentration of the plasmid DNA was measured by using a NanoDrop (Thermo, USA).

Protoplasts were isolated from rosette leaves collected from 3- to 4-week-old Col wild type plants, and used for protoplast transfection by following the procedure with co-transfection of *35S:Luciferase* as an inner control as described previously^8,33,36,37^.

For protein subcellular location assay, plasmids of *GFP-AITRL* and *NLS-RFP* were co-transfected into the protoplasts isolated, and co-transfection of *GFP* and *NLS-RFP* was used as a control. For transcriptional activity assays, plasmids of the *LexA-Gal4:GUS* reporter, the *LD-VP* activator and the *GD-AITRL* effector or the *GD* control genes were co-transfected into protoplasts. The transfected protoplasts were incubated at room temperature for 18–22 h in darkness, then GFP and RFP florescence were examined under a fluorescent microscope (Olympus, Japan), and GUS activities were measured by using a Synergy HT fluorescence microplate reader (BioTEK, USA).

### ABA sensitivity assays

ABA inhibited seed germination and seedling greening were assayed as described previously^30,38,39^. Briefly, Surface sterilized seeds of the Col wild type, the *35S:AITRL* transgenic plants and the *aitrl* mutants were sown on plates with solidified 0.5 × MS salts in the presence or absence 0.5 µM ABA, kept at 4 °C and in darkness for 3 days, and then transferred to a growth room. Germinated seeds were counted every 12 h after the transfer. Pictures were taken 12 days after the transfer, and seedlings with green cotyledons were counted. The experiments were repeated at least three times.

### Data analysis

Data obtained was analysis in Excel, and student *t *test (https://www.graphpad.com/quickcalcs/ttest1/) was used for statistic analysis.

## Results

### DRG is an AITR-like protein

In the process to identify novel players in regulating ABA signaling as described previously^8^, we found that the expression level of *DRG*, a gene previously reported to regulate drought and freezing tolerance in Arabidopsis^28^, was increased in response to ABA treatment^38^. Protein homologs analysis for DRG on Phytozome (https://phytozome.jgi.doe.gov/pz/portal.html#) shows that its most closely related protein in Arabidopsis is AITR5, a member of the AITRs family that have been show to play a feed-forward regulating role in ABA signaling^8^.

**Figure 1:**
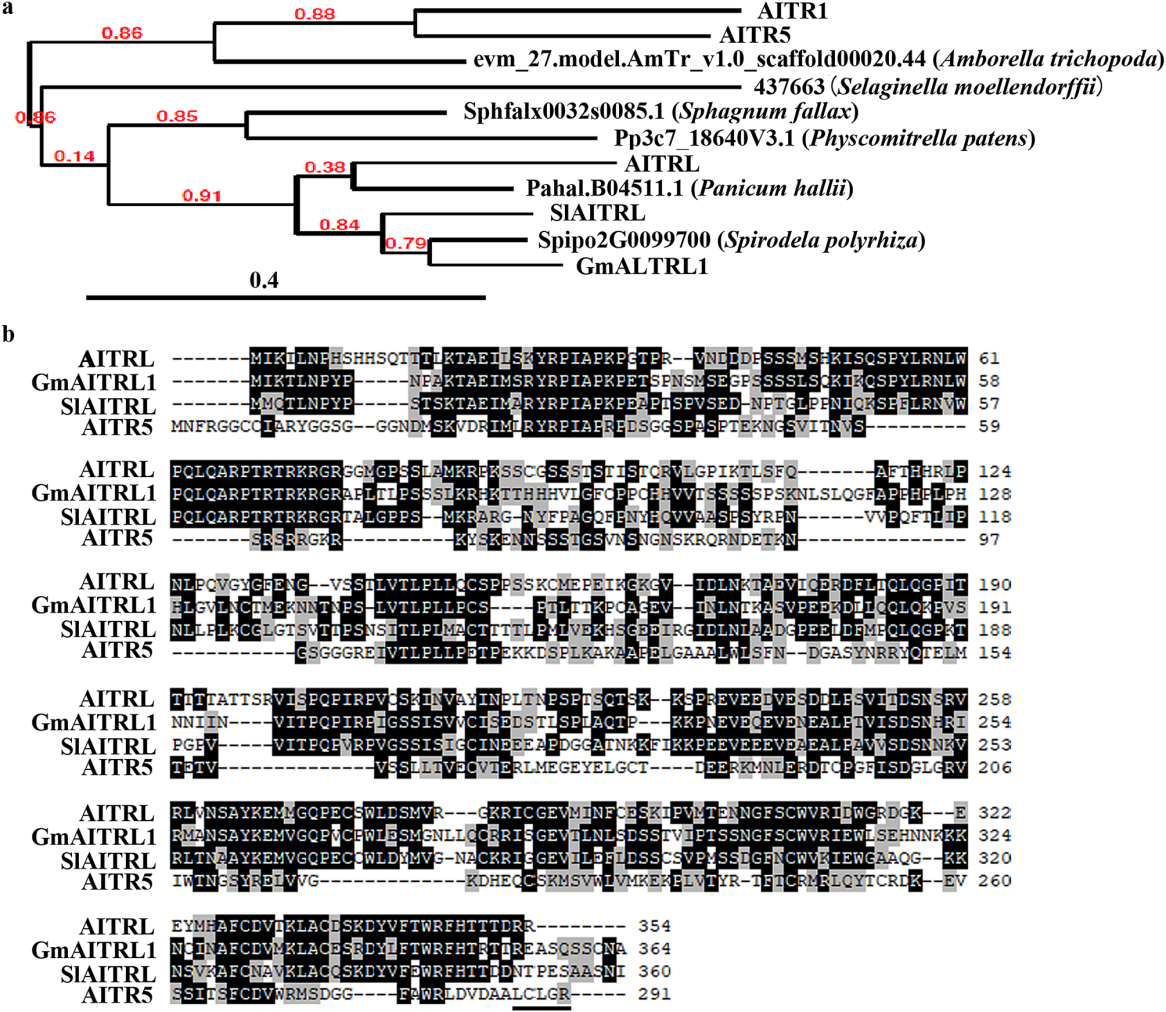
Phylogenetic analysis of AITRs and AITRLs, and amino acid alignment of AITRL, GmAITRL1, SlAITRL and AITR5. (**a**) Phylogenetic analysis of AITRs and AITRLs. AITRs and AITRLs in the plant species selected were identified on phytozome v12 (https://phytozome.jgi.doe.gov/pz/portal.html#), and their full-length amino acid sequences were used for phylogenetic analysis by using “One Click” mode on phylogeny (http://www.phylogeny.fr/simple_phylogeny.cgi) with default settings. Branch support values are indicated above the branches. (**b**) Amino acid alignment of AITRL, GmAITRL1, SlAITRL and AITR5. Full-length amino acid sequences of AITRL, GmAITRL1, SlAITRL and AITR5 were obtained on phytozome and used for sequence alignment by using BioEdit 7.0 (https://bioedit.software.informer.com/7.0/). The identical amino acids in AITRL, GmAITRL1, SlAITRL and AITR5 were shaded in black, and the similar ones in gray. Underline indicates the partial conserved LxLxL motif in AITR5.

Our previously results showed that AITRs are a family of novel transcription repressors conserved in angiosperms, and may have been evolved from AITR-like proteins found in bryophytes and gymnosperms^8^. Phylogenetic analysis shows that DRG and its homologs in both soybean and tomato, two dicot species that are either less or more closely related to Arabidopsis in the phelogenetic analysis^8^, rather than AITR5, are in the clade formed by the AITR-like proteins and AITRs from early evolved angiosperms (Fig. 1a). These results suggested that DRG is more closed related to AITR-like proteins, rather than AITR5, therefore we renamed DRG as AITRL. Amino acid alignment shows that AITRL shares high amino acid identity and similarity with its homologs from soybean and tomato (Fig. 1b), but less with AITR5. Amino acid sequence BLAST on NCBI (https://blast.ncbi.nlm.nih.gov) shows that AITRLs are plant specific proteins, and gene ancestry analysis for AITRL on Phytozome (https://phytozome.jgi.doe.gov/pz/portal.html#) shows that AITRLs are present only in embryophytes. These results indicate that AITRLs are a novel family of proteins.

### Expression of *AITRL* is up-regulated by ABA

**Figure 2:**
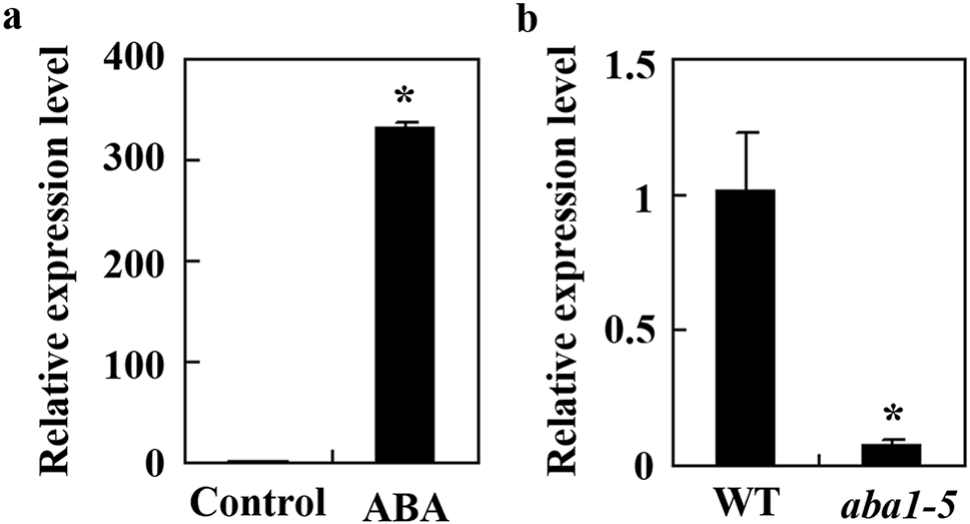
Expression of *AITRL* in response to ABA treatment. (**a**) Expression of *AITRL* in response to ABA. Twelve-day-old Col wild type seedlings were treated with 50 µM ABA or solvent methanol as a control for 4 h. Total RNA was isolated and qRT-PCR was used to examine the expression of *AITRL*. The expression of *ACT2* was used as an inner control, and the expression level of *AITRL* in the control sample was set as 1. Data represent the mean ± SD of three replicates. *Significantly different from the control (p < 0.0001). (**b**) Expression of *AITRL* in the *aba1-5* mutants. Total RNA was isolated from 12-day-old L*er* wild type and *aba1-5* mutant seedlings, and qRT-PCR was used to examine the expression of *AITRL*. The expression of *ACT2* was used as an inner control, and the expression level of *AITRL* in the wild type seedlings was set as 1. Data represent the mean ± SD of three replicates. *Significantly different from the wild type (p < 0.005).

Considering that AITRs are involved in the regulating of ABA signalling^8^, it is likely that AITRL may also play a role in regulating plant response to ABA. To examine if that is the case, we first wanted to confirm the expression of *AITRL* is regulated by ABA. To do that, total RNA was isolated from Arabidopsis seedlings treated with ABA, and used to examine the expression of *AITRL* by RT-PCR*.* As shown in Fig. 2a, the expression level of *AITRL* in Arabidopsis seedlings increased more than 300 folds in response to ABA treatment, indicating that similar to *AITRs*, *AITRL* is an ABA response gene. To further examine if *AITRL* is an ABA responsive gene, we examined the expression level of *AITRL* in the ABA deficient mutant *aba1-5*, and a more than tenfold decrease was observed in the *aba1-5* mutant seedlings when compared with the wild type seedlings (Fig. 2b).

### AITRL is a transcription repressor

**Figure 3:**
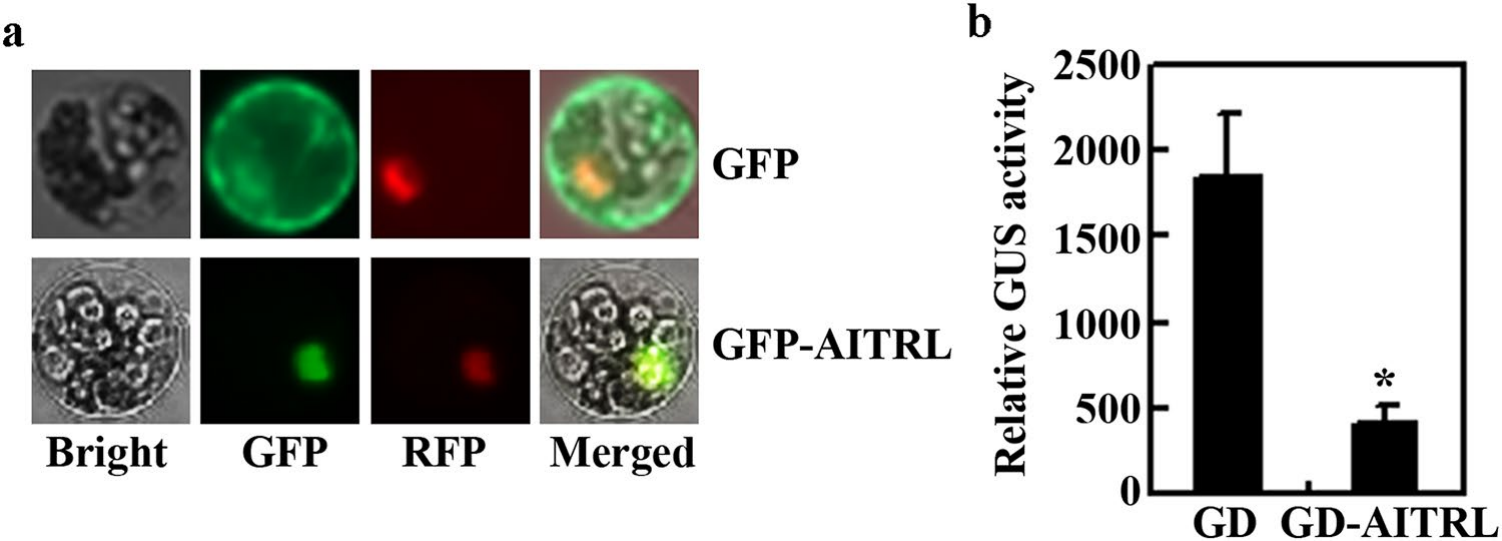
AITRL is a transcription repressor. (**a**) Subcellular localization of AITRL. Plasmids of the *GFP-AITRL* effector gene and the *NLS-RFP* nuclear marker gene were co-transfected into Arabidopsis protoplasts isolated from leaves of the Col wild type. The transfected protoplasts were incubated in darkness at room temperature for 16–18 h. The GFP and RFP fluorescence was observed under a fluorescence microscope. Co-transfection of *GFP* effector gene and the *NLS-RFP* nuclear marker gene was use as a control. (**b**) Transcriptional activities of AITRL. Plasmids of the *LexA-Gal4:GUS* reporter gene, the *LD-VP* transcription activator gene and the *GD-AITRL* effector gene were co-transfected into Arabidopsis protoplasts isolated from leaves of the Col wild type plants. Co-transfection of the *GD* effector gene was used as a control. The transfected protoplasts were incubated in darkness at room temperature for 20–22 h. GUS activities were measured by using a microplate reader. Data represent the mean ± SD of three replicates. *Significantly different from the GD (p < 0.005).

After confirmed that the expression of *AITRL* was induced by ABA (Fig. 2), we examined if AITRL may functions as a transcription repressor. It has been shown that AITRL is a nuclear protein^28^. To confirm this by using Arabidopsis protoplast transit transfection assays, plasmid DNA of *GFP-AITRL* was transfected into Arabidopsis protoplasts, and GFP fluorescence was observed under a cofocal microscope. As shown in Fig. 3a, GFP fluorescence was specifically observed in the nucleus. As a control, no specific subcellular localization was observed for GFP alone (Fig. 3a).

Arabidopsis protoplast transit transfection assays were also used to examine the transcriptional activities of AITRL. Plasmids of the *LexA-Gal4:GUS* reporter, the *LD-VP* activator and the *GD-AITRL* effector or the *GD* control genes were co-transfected into Arabidopsis protoplasts, and GUS activities were measured by using a microplate reader. As shown in Fig. 3b, GUS activity activated by the LD-VP activator was repressed by the co-transfection of *GD-AITRL*, suggesting that AITRL functions as a transcription repressor.

### The *aitrl* mutants are hypersensitive to ABA

Having shown that the expression of *AITRL* was induced by ABA and AITRL functions as a transcription repressor, we further examined the function of AITRL in regulating ABA response by using ABA inhibited seed germination and seedling greening assays.

**Figure 4:**
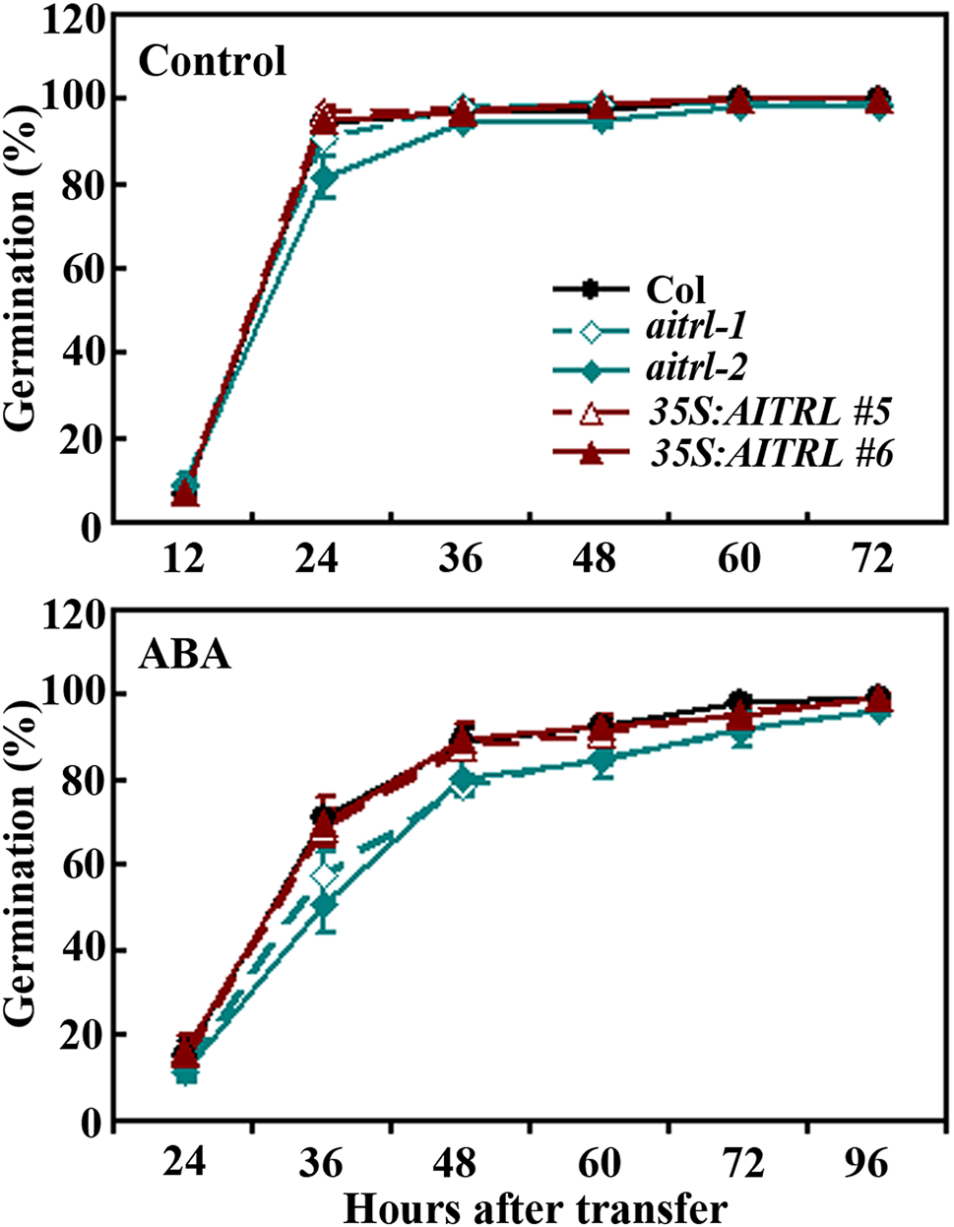
Effects of ABA on seed germination of the Col wild type, the *35S:AITRL* transgenic plants and the *aitrl* mutants. Seeds of the Col wild type, the *35S:AITRL* transgenic plants and the *aitrl* mutants were sterilized and sown on solidified 0.5 × MS salts plates in the presence or absence of 0.5 µM ABA. The plates were kept at 4 °C in darkness for 3 days before transferred to a growth room. Germinated seeds were counted every 12 h after the transfer till all the seeds were germinated, and percentage of seed germination was calculated. Data represent the mean ± SD of three replicates.

In seed germination assays, we found that even though slightly reduced germination rate was observed for the seeds of the *aitrl* mutants 24 h after the plates were transferred into a growth room, all the seeds including that of the Col wild type, the *35S:AITRL* transgenic plants, and the *aitrl* mutants on the control plates germinated 36 h after the transfer (Fig. 4). However, on the ABA-containing plates, when compared with seeds of the Col wild type plants, lower germination rate for seeds of the *aitrl* mutants was observed at most of the time points examined (Fig. 4), indicating that the *aitrl* mutants are more sensitivity to ABA treatment.

**Figure 5:**
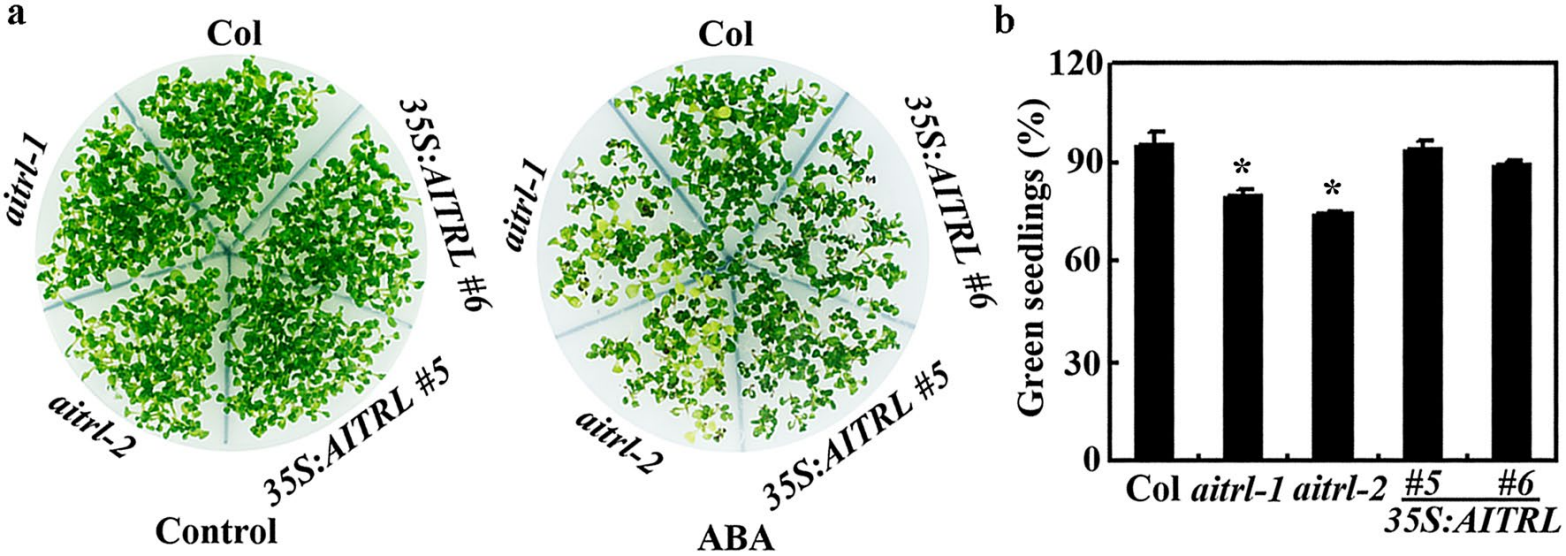
Effects of ABA on seedling greening of the Col wild type, the *35S:AITRL* transgenic plants and the *aitrl* mutants. (**a**) Seedling greening of the Col wild type, the *35S:AITRL* transgenic plants and the *aitrl* mutants in response to ABA treatment. Seeds were sterilized and sown on plates with solidified 0.5 × MS salts in the presence or absence 0.5 µM ABA. The plates were kept at 4 °C in darkness for 3 days before transferred to a growth room. Pictures were taken 12 days after the transfer. (**b**) Percentage of green seedlings of the Col wild type, the *35S:AITRL* transgenic plants and the *aitrl* mutants in response to ABA treatment. Seedlings with green cotyledons were counted 12 days after the transfer, and the percentage of green seedlings was calculated. Data represent means ± SD of three replicates. *Significantly different from the wild type (p < 0.05).

Similar, increased sensitivity to ABA treatment in the *aitrl* mutants was observed in seedling greening assays (Fig. 5a). Quantitative assays showed that the green cotyledon rate of the *aitrl* mutant plants was ~ 75%, compared with ~ 95% of that in the Col wild type plants (Fig. 5b). On the other hand, the green cotyledon rate of the *35S:AITRL* transgenic plants was largely indistinguishable from that of the Col wild type plants (Fig. 5b).

### Expression of ABA genes was affected in the *aitrl* mutants

**Figure 6:**
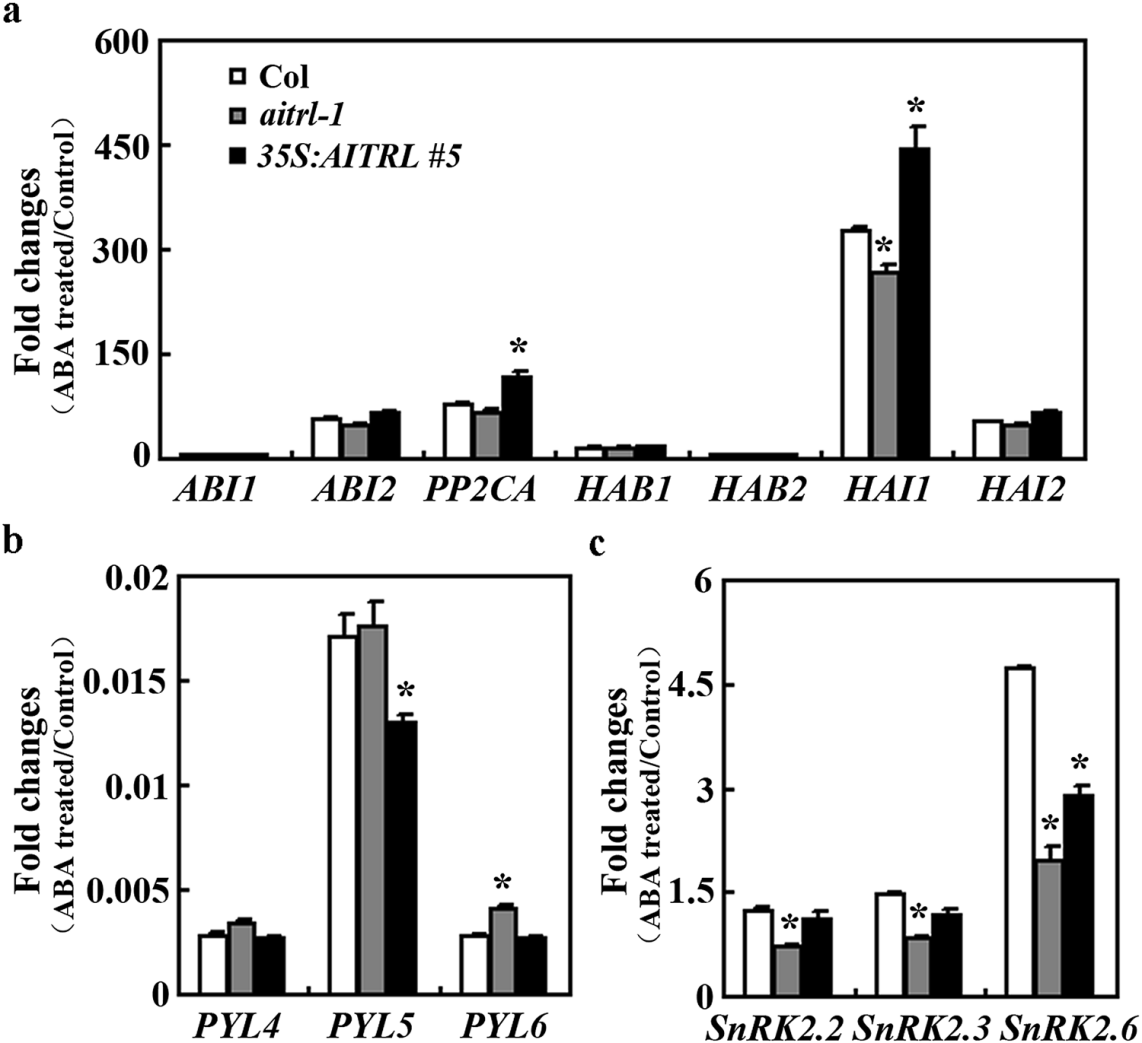
Expression of ABA signaling component genes in the Col wild type, the *35S:AITRL* transgenic plants and the *aitrl* mutants. Expression of *PP2C* genes (**a**), *PYL* genes (**b**) and *SnRK2* genes (**c**) in response to ABA in the Col wild type, the *35S:AITRL* transgenic plants and the *aitrl* mutants. Twelve-day-old seedlings were treated with 50 µM ABA or solvent methanol as a control for 4 h. Total RNA was isolated and qRT-PCR was used to examine the expression of the ABA signaling component genes. The expression of *ACT2* was used as an inner control. Fold changes were calculated by comparing the expression levels of the corresponding genes in ABA treated and the control seedlings. Data represent the mean ± SD of three replicates. *Significantly different from the wild type (p < 0.05).

We have previously shown that AITRs regulate the expression of some ABA signaling component genes^8^, having shown that ABA sensitivity was increased in the in *aitrl* mutants (Figs. 4, 5), we then examined if the expression of ABA signaling component genes was regulated by AITRL. As expected, the expression of some *PP2C* genes was dramatically induced by ABA in the Col wild type seedlings, however, the fold changes of the expression of the *PP2C* gene *HAI1* in response to ABA were reduced in the *aitrl* mutants, and increased in the *35S:AITRL* transgenic plant seedling (Fig. 6a). On the other hand, the expression of *PYL* genes including *PYL4*, *PYL5* and *PYL6* was dramatically repressed by ABA in the Col wild type seedlings, however, decrease in ABA inhibition of the expression of *PYL6* was observed in the *aitrl* mutants, whereas enhance of *PYL5* was observed in the *35S:AITRL* transgenic plant seedling (Fig. 6b). We also observed that ABA induced expression of some *SnRK2* genes including *SnRK2.2*, *SnRK2.3* and *SnRK2.6* was reduced in the *aitrl* mutant seedlings, but also reduced or remained unchanged in the *35S:AITRL* transgenic plant seedling (Fig. 6c). These results suggest that AITRL may regulate ABA responses in Arabidopsis by regulating the expression of ABA signaling component genes.

### AITRLs are evolutionarily conserved in plants

**Figure 7:**
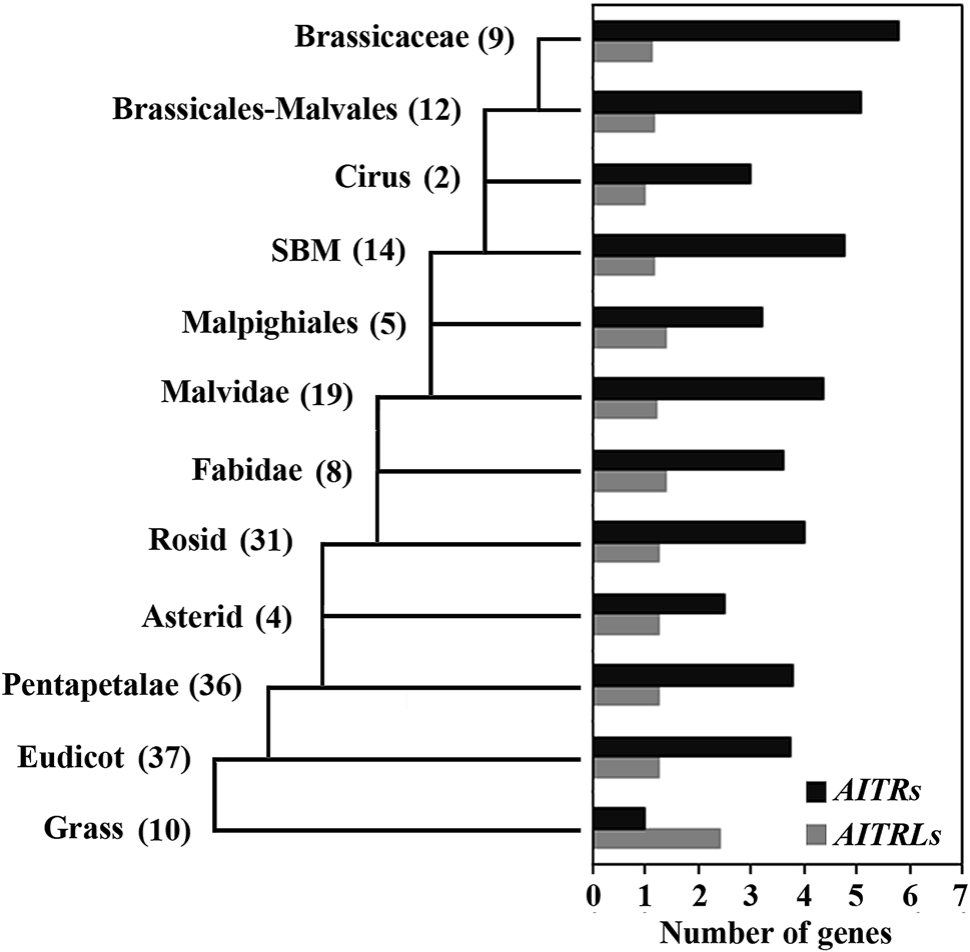
Changes of *AITRs* and *AITRLs* gene numbers during evolution of angiosperm. Numbers of *AITRs* and *AITRLs* genes were obtained by using *AITR5* and *AITRL* respectively, for “Gene Ancestry” assays on phytozome. Average gene numbers per plant specie were calculated for different catalogs of angiosperm. Numbers in the brackets indicate total plant species available on phytozome in the indicated catalogs.

Similar to *AITRs*, the expression of *AITRL* was induced by ABA (Fig. 2), and AITRL functions as a transcription repressor (Fig. 3). However, unlike the *aitrs* mutants which showed a reduced ABA sensitivity^8^, ABA sensitivity was increased in the *aitrl* mutants (Figs. 4, 5), and opposite effects for AITRL and AITRs on the expression of ABA signaling component genes were also observed^8^ (Fig. 6). Considering that it is likely that AITRs are evolved from AITRLs^8^, and AITRLs are presented in most recently evolved plants including Arabidopsis (Fig. 1), we examined the distribution of *AITRL* and *AITR* genes in angiosperms. We found that grass plants have an average of more than 2 *AITRL* genes, but only 1 *AITR* gene, while eudicot plants have 1 *AITRL* gene, but nearly 4 *AITR* genes (Fig. 7). We also found that the number of *AITRL* genes remained largely unchanged, however, the number of *AITR* genes increased slowly during the evaluation process of eudicot plants, as a result, brassicassaes have an average of more than 6 *AITR* genes (Fig. 7).

## Discussion

We have previously shown that AITRs are a family of novel transcription repressors that play a feed-forward regulating role in ABA signaling, AITRs may evolved from AITR-like proteins in bryophytes and gymnosperms and are conserved in angiosperms^8^. We identified here *DRG* as an ABA inducible *AITRL* gene, we found that AITRL plays a role in regulating ABA responses, and AITRLs are an evolutionally conserved family of novel transcription repressors in embryophytes.

Expression of stress related genes can be regulated by ABA dependent and independent pathways^1,9–11,26,27^. *DRG* has previously been reported to be a drought response gene^28^, we found that its expression was up-regulated by ABA (Fig. 2). Phylogenetic analysis shows that AITRL is closely related AITR-like proteins in bryophytes and gymnosperms (Fig. 1), amino acid sequence BLAST results show that AITRLs are plant specific proteins, and are evolutionally conserved in embryophytes (Fig. 7).

Even though both *AITRL* and *AITRs* are up-regulated by ABA^8^ (Fig. 2), and similar to AITRs^8^, AITRL functioned as a transcription repressor (Fig. 3), our results show that AITRL and AITRs have opposite functions in regulating ABA responses in Arabidopsis. In both the seed germination and seedling greening assays, the *aitrl* mutants showed an increased sensitivity to ABA (Figs. 4, 5), whereas *aitrs* mutants showed a decreased sensitivity to ABA^8^. In addition, ABA responses of some ABA signaling component genes were reduced the *aitrl* mutants (Fig. 6), but increased in the *aitrs* mutants^8^. Consistent with their opposite functions in regulating ABA response, *drg*/*aitrl-1* mutants showed a decreased tolerance to abiotic stresses including drought and freezing^28^, whereas *aitrs* mutants showed enhanced tolerance to abiotic stresses such as drought and salt stresses^8^. Considering that both AITRL and AITRs are presented in angiosperms, it is very likely that antagonism functions of AITRLs and AITRs in regulating ABA signaling in plants fine turn the plant responses to abiotic stresses. It is should be noted that closely related transcriptional regulators have opposing effects in regulating ABA signaling and plant responses to abiotic stresses have been observed in different transcription factor families. For example, both of the bHLH transcription factor genes *ABA-INDUCIBLE BHLH-TYPE TRANSCRIPTION FACTOR* (*AIB*) and *ANDROGEN-INDUCIBLE GENE 1* (*AtAIG1*) are ABA response genes, but AIB positively, and AtAIG1 negatively regulate ABA response^40,41^. Whereas the ERF transcription factor AP2-like ABA repressor 1 (ABR1) and AtERF15 also have opposing effects in regulating both ABA response and abiotic stress response in Arabidopsis^42,43^.

As discussed previously, the appearance of AITRs during evolution may lead to reduced abiotic stress tolerance in plants^8^. Our evolutionary comparison analysis showed that there are more *AITRL* genes in grass plants, but more *AITR* genes in eudicot plants, and the numbers of *AITRLs* remained largely unchanged, where as that of AITRs increased during the evolution of eudicots (Fig. 7), further suggest that reduced tolerance in plants during evolution may be caused by the appearance of *AITRs*, and that AITRLs may play a positive role in regulating plant tolerance to abiotic stresses.

As mentioned above, both *AITRL* and *AITRs* are ABA responsive transcription repressor genes, but have opposite functions in regulating plant responses to ABA and abiotic stresses, and ABA response of some ABA signaling component genes. We noted that AITRs have a full or partial conserved LxLxL motif at the C-terminal^8^, an ERF-associated amphiphilic repression (EAR) repression motif initially identified in class II ERFs ^44^. However, AITRL does not (Fig. 1).

It has been shown that EAR motif-containing proteins mediated transcriptional repression represent the main form of transcriptional repression in plants^45,46^. They can mediate transcription repression in at least two different ways^45^, one is epigenetic modification by recruiting a histone deacetylase (HDAC) and interacting with co-suppressors to form a HDAC complex. For example, ERF7 can interact with SIN3 to recruit HDA19 to form a HDAC complex^47^. Another is, similar to other transcription repressors, interference of the activities of other transcription factors via directly or indirectly binding. For example, OFP1 and OFP4 interact with KNAT7 to enhance its repression activities^48^. It is likely that the different functions observed for AITRL and AITRs may be caused by different ways they are mediating transcriptional repression. Never the less, it will be of interest to examine if the L × L × L motif is responsive for the different functions observed for AITRL and AITRs, therefore to figure out how AITRL may mediate transcription repression.

ABA signaling lead to the activation of ABF/AREB/ABI5-type bZIP transcription factors, which in turn activate/repress the expression of ABA response genes^4,5,13,14,18–20^. We showed that the expression of *AITRL* was up-regulated by ABA (Fig. 2), it is worthwhile to examine if the expression of *AITRL* is regulated by ABF/AREB/ABI5-type bZIP transcription factors. On the other hand, the expression of *AITRL* was up-regulated by drought^28^. Considering that NAC and AP2/ERF transcription factor DREB1 and DREB2 are able to activate drought response genes via directly binding to the DRE *cis*-element^26,27^, it will be also of interest to examine if DREB1 and DREB2 may regulate the expression of *AITRL* in an ABA independent way.

In summary, we found that *AITRL* is an ABA response gene, AITRL negatively regulates ABA responses in Arabidopsis, AITRLs are a novel family of transcription repressors conserved in embryophytes, and antagonism functions of the AITRLs and AITRs in regulating ABA signaling in plants may fine turn the plant responses to abiotic stresses.
